# Impact of Audio Data Compression on Feature Extraction for Vocal Biomarker Detection: Validation Study

**DOI:** 10.2196/56246

**Published:** 2024-04-15

**Authors:** Jessica Oreskovic, Jaycee Kaufman, Yan Fossat

**Affiliations:** 1 Klick Labs Toronto, ON Canada

**Keywords:** vocal biomarker, biomarker, biomarkers, sound, sounds, audio, compression, voice, acoustic, acoustics, audio compression, feature extraction, Python, speech, detect, detection, algorithm, algorithms

## Abstract

**Background:**

Vocal biomarkers, derived from acoustic analysis of vocal characteristics, offer noninvasive avenues for medical screening, diagnostics, and monitoring. Previous research demonstrated the feasibility of predicting type 2 diabetes mellitus through acoustic analysis of smartphone-recorded speech. Building upon this work, this study explores the impact of audio data compression on acoustic vocal biomarker development, which is critical for broader applicability in health care.

**Objective:**

The objective of this research is to analyze how common audio compression algorithms (MP3, M4A, and WMA) applied by 3 different conversion tools at 2 bitrates affect features crucial for vocal biomarker detection.

**Methods:**

The impact of audio data compression on acoustic vocal biomarker development was investigated using uncompressed voice samples converted into MP3, M4A, and WMA formats at 2 bitrates (320 and 128 kbps) with MediaHuman (MH) Audio Converter, WonderShare (WS) UniConverter, and Fast Forward Moving Picture Experts Group (FFmpeg). The data set comprised recordings from 505 participants, totaling 17,298 audio files, collected using a smartphone. Participants recorded a fixed English sentence up to 6 times daily for up to 14 days. Feature extraction, including pitch, jitter, intensity, and Mel-frequency cepstral coefficients (MFCCs), was conducted using Python and Parselmouth. The Wilcoxon signed rank test and the Bonferroni correction for multiple comparisons were used for statistical analysis.

**Results:**

In this study, 36,970 audio files were initially recorded from 505 participants, with 17,298 recordings meeting the fixed sentence criteria after screening. Differences between the audio conversion software, MH, WS, and FFmpeg, were notable, impacting compression outcomes such as constant or variable bitrates. Analysis encompassed diverse data compression formats and a wide array of voice features and MFCCs. Wilcoxon signed rank tests yielded *P* values, with those below the Bonferroni-corrected significance level indicating significant alterations due to compression. The results indicated feature-specific impacts of compression across formats and bitrates. MH-converted files exhibited greater resilience compared to WS-converted files. Bitrate also influenced feature stability, with 38 cases affected uniquely by a single bitrate. Notably, voice features showed greater stability than MFCCs across conversion methods.

**Conclusions:**

Compression effects were found to be feature specific, with MH and FFmpeg showing greater resilience. Some features were consistently affected, emphasizing the importance of understanding feature resilience for diagnostic applications. Considering the implementation of vocal biomarkers in health care, finding features that remain consistent through compression for data storage or transmission purposes is valuable. Focused on specific features and formats, future research could broaden the scope to include diverse features, real-time compression algorithms, and various recording methods. This study enhances our understanding of audio compression’s influence on voice features and MFCCs, providing insights for developing applications across fields. The research underscores the significance of feature stability in working with compressed audio data, laying a foundation for informed voice data use in evolving technological landscapes.

## Introduction

### Background

Vocal biomarkers are emerging as a promising accessible and noninvasive avenue for medical screening, diagnostics, and monitoring [1]. These biomarkers are unique characteristics or acoustic patterns of an individual’s voice that can hold valuable information about their physical and mental well-being [2]. Human voice production requires the coordination of multiple biological systems; perturbations in these systems induced by various conditions or diseases can result in alterations in the characteristics of the human voice [3]. Potential applications of vocal biomarkers are diverse, including the identification of neurological disorders, cardiovascular diseases, respiratory conditions, and mental health disorders, among others [2,4-6].

In our previous work, “Acoustic Analysis and Prediction of Type 2 Diabetes Mellitus Using Smartphone-Recorded Voice Segments” [7], smartphone-recorded speech was used to predict type 2 diabetes mellitus through a comprehensive acoustic analysis [7]. The study demonstrated the feasibility of using acoustic features from smartphone-recorded voice data to predict the presence of this disorder, highlighting the valuable diagnostic potential of vocal biomarkers in the context of a specific health condition [7]. Building upon this prior research, we aim to assess the impact of audio compression on acoustic vocal biomarker development, which is crucial for the broader applicability of this emerging field.

The development of acoustic vocal biomarkers relies on the analysis of voice data, and this process is multifaceted. One critical aspect of this analysis is feature extraction, which involves identifying and quantifying relevant acoustic features from the voice data [8]. These features may encompass a wide range of parameters such as pitch, spectral properties, prosodic patterns, and various other characteristics that carry meaningful information about the speaker’s health status [2,8]. Accurate and robust feature extraction is pivotal for the successful identification and interpretation of vocal biomarkers.

Voice data are often captured, transmitted, and stored in various digital formats that may include compression, a common practice used to reduce the size of audio files, making them more manageable and efficient for storage and transmission [9]. It is necessary to consider the potential impact of audio data compression on the overall process of vocal biomarker development as the process can have significant effects on the audio [10]. Compression algorithms are widely applied to raw, high-quality audio (typically waveform audio file format) and can be classified as lossy or lossless [11]. Lossy compression algorithms reduce file size to as low as 10% of the original size by removing mostly inaudible audio data, while lossless preserves all the original audio data and only compresses to approximately 50% [12]. Some of the most common lossy formats include MP3, M4A, and WMA [12]. These formats offer different trade-offs between file size and audio quality, and each may introduce specific artifacts and alterations to the original acoustic data.

Previous research on how data compression impacts voice signals has found that different microphones and MP3 compression bitrates on sustained vowel sounds can significantly affect feature values [10]. Research has found that various digital platforms and their audio codecs affect the voice in a way that challenges voice recognition processes specifically by narrowing the frequency band and centrally shifting frequencies at the upper and lower limits [13]. While differing microphones can introduce differences in audio data depending on specifications, smartphone microphones have been found to collect high-quality audio data suitable for acoustic analysis [14].

This exploratory research aims to investigate the effect of common audio data compression algorithms, such as MP3, AAC (compression algorithm for M4A), and WMA, on the vocal biomarker feature extraction process. Additionally, the effect of compression bitrate or encoder type will be analyzed to determine whether these factors make a difference within each format. Understanding the impact of popular data compression methods on acoustic vocal biomarker analysis is important as it can significantly affect the quality and interpretability of biomarker data [15,16]. Moreover, this knowledge can guide the development of best practices and inform the compression implementation process for the specific needs of health care applications, such as remote medical care involving telephone or video conferencing, thereby minimizing the risk of unintentional distortion of vocal biomarkers.

### Objective

The objective of this research is to analyze the effect of several common audio data compression algorithms: MP3, M4A, and WMA, in 2 common bitrates, completed by 3 different conversion tools, on feature extraction from voice data for vocal biomarker detection.

## Methods

### Overview

In this research, acoustic features were derived from uncompressed voice samples, which were subsequently converted into MP3, M4A, and WMA formats using 3 distinct tools, namely MediaHuman (MH) Audio Converter, WonderShare (WS) UniConverter, and Fast Forward Moving Picture Experts Group (FFmpeg) across 2 different bitrates (320 and 128 kbps). MH, WS, and FFmpeg conversion tools were selected because of their accessibility as free, downloadable audio conversion software. Our goal was to explore how different audio conversion tools, formats, and 2 specific bitrates affect the data set used to develop a biomarker prediction model [7]. By focusing on these tools and bitrates, we aimed to provide insights into the potential impact of common audio compression methods on the extracted voice features. This approach allowed for a manageable analysis while paving the way for future research to delve deeper into the nuances of audio compression effects on biomarker prediction models.

### Data and Participants

This research was conducted using a data set of audio recordings that were collected from 505 participants (mean age 41.03, SD 13.29 years, 336 male participants) recruited between August 30, 2021, and June 30, 2022, for a study in India [7]. Participants were instructed to record a short English phrase up to 6 times daily using their smartphone for 14 consecutive days. As these data were originally recorded for research involving diabetes, the phrase was “Hello. How are you? What is my glucose level right now?” All audio files used in the research originated in the uncompressed waveform audio file format, 16-bit 44.1 kHz.

Participants in this study used a variety of smartphone models for data recording. While efforts were made to request recordings in quiet environments, the inherent difficulty in controlling recording conditions may have introduced variability in the recorded speech data. No preliminary tests were conducted to assess the recording quality across different smartphone models, and no preprocessing techniques were applied to address potential hardware variations in the recorded speech data. It is noteworthy that the intention of the prediction model was to be run on a smartphone; therefore, the recordings were made using smartphone uncompressed audio to align with the intended application context.

### File Conversion

To explore the impact of diverse data compression methods, the original files underwent conversion using MH (version 2.2.2), WS (version 15), and FFmpeg (version 6.1.1) in Python (version 3.10.11; Python Software Foundation) on a PC. Three distinct compression algorithms—MP3, M4A, and WMA—at 2 bitrates—128 kbps and 320 kbps—were applied to simulate real-world scenarios where audio data are commonly subjected to different compression algorithms for storage and transmission purposes. The sample rate (44.1 kHz) and the channels (stereo) were kept consistent over all formats. The choice of encoders used in the research was not a primary consideration; rather, our focus was on comparing the results obtained from different compression methods. It is worth noting that the selected encoders were accessible, free, and capable of batch processing multiple files, which facilitated efficient experimentation. Despite maintaining consistency in factors such as bitrate, channels, and formats between the 3 encoders, there are features of the tools that remain hidden that could potentially cause differences in the converted files, such as the encoding mode (ie, constant or variable bitrate) or other encoding options. However, these hidden features are not a large concern because the objective of the study was to compare compressed and uncompressed data rather than comparing between compression. The incorporation of multiple encoders served the purpose of discerning whether factors beyond just bitrate and file format influenced feature values.

### Feature Extraction and Comparison

We chose to use the same feature set (Multimedia Appendix 1) as in our previous research on developing a voice-to-type 2 diabetes model to maintain consistency and leverage their established effectiveness in capturing relevant biological information from voice data [7]. Acoustic features were extracted from both the original waveform audio file format files and the compressed audio formats using Python (version 3.10.11; Python Software Foundation). The voice feature extraction process leveraged Parselmouth, a Python integration of Praat speech and voice analysis software [17,18], ensuring robustness and accuracy in feature extraction. The extracted features aimed to capture pertinent acoustic characteristics of the voice data, such as pitch, jitter, and intensity, as well as Mel-frequency cepstral coefficients (MFCCs) [19], which have demonstrated efficacy in capturing subtle variations in vocal properties associated with health conditions.

Notable perceived voice qualities such as breathiness, hoarseness, and roughness, which typically present with elevated levels of shimmer and jitter, were often associated with certain pathological conditions and were therefore included in the biomarker development as well as this research [7,20]. While acoustic analysis is mainly performed using sustained phonation of vowel sounds, recent studies have demonstrated the use of shimmer and jitter measurements in identifying dysphonia even when calculated from entire sentence recordings [20]. Thus, because the data set was originally studied for the purpose of biomarker development, we chose to include the evaluations of shimmer and jitter alongside traditional vocal parameters such as pitch, intensity, and harmonic noise ratio in this analysis of how audio data compression impacts feature values.

Given the non-Gaussian distribution of feature data, assessed via the Shapiro-Wilk test, a nonparametric approach—specifically, the Wilcoxon signed rank test—was adopted for statistical analysis. This paired test aimed to evaluate the impact of each compression method on audio features by comparing the features extracted from the original uncompressed files with those obtained from each compressed format individually. In this study, the Bonferroni correction method was used to account for multiple comparisons. Given our focus on assessing the impact of each conversion method relative to the original feature values rather than comparing between different treatments, this correction was deemed appropriate. This approach allowed us to effectively manage the potential for false positives while evaluating the stability of feature values across different compression methods.

### Ethical Considerations

The protocol (ID MGCTS107) received ethics approval by Saanvi Ethical Research LLP, all participants signed informed consent, and data were stored in a secure cloud database with no identifying information. Participants were compensated for their time.

## Results

### Data and Participants

A total of 36,970 audio files were recorded from the 505 participants who completed the study. Speech-to-text screening ensured that the audio files adhered to the fixed sentence criteria and were devoid of substantial background noise, resulting in a total of 17,298 recordings. All participants were native to India.

### File Conversion

The noncustomizable differences between the audio conversion software MH Audio Converter and WS UniConverter manifested in evident variations in the converted files. Table 1 displays the differences in compression ratio and data set size, highlighting these distinctions and emphasizing the impact of software-specific characteristics on the compression outcomes such as constant or variable bitrates.

**Table 1:** Compression specifications for each data compression method including the final size of the data set and compression ratio.

Format and tools		Bitrate (kbps)		Data set size (GB)		Compression ratio
**MP3**						
	MediaHuman	128 320	1.29 3.22		5.42 2.17	
	WonderShare	128 320	0.43 1.05		16.26 6.66	
	FFmpeg^a^	128 320	1.29 3.20		5.42 2.18	
**M4A**						
	MediaHuman	128 320	1.39 5.10		5.03 1.37	
	WonderShare	128 320	0.47 1.10		14.87 6.35	
	FFmpeg	128 320	1.26 2.18		5.55 3.21	
**WMA**						
	MediaHuman	128 320	1.39 5.49		5.03 1.27	
	WonderShare	128 320	0.70 1.33		9.99 5.26	
	FFmpeg	128 320	1.39 5.49		5.03 1.27	

^a^FFmpeg: Fast Forward Moving Picture Experts Group.

### Feature Extraction and Comparison

This research investigated the influence of diverse data compression formats on an extensive array of voice features and MFCCs. The corresponding *P* values for each feature are provided in the subsequent table from the results of the 756 Wilcoxon signed rank tests. *P* values below the level of significance, 6.61×10^–5^ with the Bonferroni correction (Table S1-S3 in Multimedia Appendix 2), signify a notable difference in feature values between the original .wav format and the corresponding compressed format, indicating a significant alteration due to compression. Conversely, features with *P* values greater than 6.61×10^–5^ (Table S1-S3 in Multimedia Appendix 2) are deemed robust, suggesting their resilience to the compression process.

## Discussion

### Overview

This investigation illuminated the effects of diverse audio file compression methods on a broad spectrum of voice features and MFCCs. The results revealed that the impact of data compression is feature specific and varies across different encoders, formats, and bitrates.

### Principal Findings

The encoder played a substantial role in influencing voice features, with MH- and FFmpeg-converted files demonstrating greater resilience to compression compared to WS-converted files, regardless of the format. For MH, WS, and FFmpeg, there were 15, 6, and 21 features, respectively, that had at least 1 format or bitrate combination that was unaffected by the conversion. A total of 59 compressed feature comparisons showed stability for MH, 8 for WS, and 67 for FFmpeg (Table S1-S3 in Multimedia Appendix 2). The conversion bitrate also exhibited an impact on feature stability, with some features remaining consistent for both bitrates, while others were affected uniquely at either 128 kbps or 320 kbps. A total of 38 feature comparison cases (of the total of 134) were only affected by compression for a single bitrate. Of those 38, 15 feature comparisons were only unaffected with 128 kbps, while 23 were stable for compression at only 320 kbps. MH and FFmpeg conversions had more features unaffected when conversions were done with a bitrate of 320 kbps compared to 128 kbps. Additionally, the voice features were found to be more stable than the MFCCs. The findings indicate that not all voice features respond equally to audio file compression. Certain features exhibited robustness and remained consistent despite compression, holding promise for applications involving compressed voice data storage or transmission. For instance, in our previous work on type 2 diabetes prediction from voice, features such as mean fundamental frequency/pitch (meanF0), pitch SD (stdevF0), and relative average perturbation jitter (rapJitter) remained consistent across several compression methods, including MP3 from MH at 320 kbps and FFmpeg at both 320 and 128 kbps and WMA from MH and FFmpeg at both 128 kbps and 320 kbps (Table S1-S3 in Multimedia Appendix 2) [7]. For the male prediction model, 1 of the 2 features (meanI) was significantly affected by all conversion methods. The second feature (apq11) remained stable for conversions with MH and FFmpeg to WMA format at both bitrates, MP3 at 320 kbps, and MH-converted M4A at 320 kbps. (Table S1-S3 in Multimedia Appendix 2) [7]. However, this study also identified features significantly altered by compression (Table S1-S3 in Multimedia Appendix 2), emphasizing the need to understand the stability and sensitivity of individual features for maintaining accuracy and interpretability in applications like health care diagnostics and voice recognition.

Vocal biomarkers, being a relatively new concept, are predominantly situated within the realm of research rather than practical settings where considerations for data storage and transmission are paramount. The study’s implications extend to various fields, particularly in health care, where voice data are increasingly used for disease detection and monitoring. When dealing with features significantly influenced by a specific compression algorithm, considerations should be made to preserve accuracy in applications requiring high diagnostic precision. The study suggests that certain voice features can withstand common data compression formats, enabling the use of compressed data in medical applications without compromising diagnostic accuracy, depending on the features. This is crucial in scenarios involving limited bandwidth for audio data transmission or storage constraints, where choosing an appropriate compression format while considering feature resilience becomes pivotal. Conversely, for research applications where features are being investigated, the use of uncompressed or lossless compression is essential.

### Limitations and Future Directions

This study has several limitations. First, while it focused on a specific set of voice features and how they were changed based on compression formats, future research could benefit from isolating compression settings to study their individual effects rigorously. Second, controlling microphone and recording settings could enhance data consistency and reliability, as variations in these factors may introduce confounding variables. Additionally, exploring different recording sentences could provide insights into how content variability influences the impact of compression on feature extraction. Finally, a broader exploration of diverse features beyond those examined in this study, such as spectral or temporal features, could offer a more comprehensive understanding of the impact of compression on acoustic vocal biomarkers.

### Conclusions

In this research, we have provided insights into the influence of audio data compression on feature values used in biomarker prediction model development. Our findings underscore the importance of considering compression effects in the design and optimization of diagnostic tools reliant on voice-based biomarkers. Through analysis and statistical comparisons, we have demonstrated the nuanced impact of compression formats, bitrates, and conversion tools on the stability and reliability of extracted feature values. By revealing these effects, our research not only advances our understanding of the complex interplay between audio data processing and biomarker extraction but also offers practical implications for health care practitioners and researchers. Moving forward, the findings pave the way for future investigations aimed at refining compression strategies, exploring alternative extraction methodologies, and ultimately enhancing the accuracy and efficacy of biomarker-based diagnostic models in clinical practice.

## Appendix

Multimedia Appendix 1 Voice feature set.

Multimedia Appendix 2 Pairwise comparisons for feature values between compressed and uncompressed audio data.

## Abbreviations

FFmpeg: Fast Forward Moving Picture Experts Group
MFCC: Mel-frequency cepstral coefficient
MH: MediaHuman
WS: WonderShare

## Data Availability

Data were commissioned by Klick Health, a private business, and are owned by Klick who does not allow sharing them.
