# Supplementary material for: Impact of Audio Data Compression on Feature Extraction for Vocal Biomarker Detection: Validation Study
Source: JMIR Biomed Eng. 2024 Apr 15;9:e56246. doi: 10.2196/56246 (PMC11058552; doi:10.2196/56246)
Supplement: Multimedia Appendix 1 [file biomedeng_v9i1e56246_app1.docx]

Voice Features

meanF0 - Mean pitch value for the voice recording.

stdevF0 - Standard deviation of the pitch for the voice recording.

meanI - Mean intensity for the voice recording.

stdevI - Standard deviation of the intensity for the voice recording.

HNR - Harmonic-to-Noise Ratio, or Harmonicity. Represents the degree of acoustic periodicity in a signal and measures the ratio between the energy in the harmonics and the noise, expressed in dB. Higher HNR values indicate more energy in the harmonics.

localShimmer - The average difference between the amplitudes of consecutive periods, divided by the average amplitude.

localdbShimmer - The average logarithm (base-10) of the difference between the amplitudes of consecutive periods, multiplied by 20.

apq3Shimmer - Amplitude Perturbation Quotient for three points. It measures the average difference between the amplitude of a period and the average amplitude of its neighboring periods, divided by the average amplitude.

apq5Shimmer - Amplitude Perturbation Quotient for five points. It determines the average difference between the amplitude of a period and the average amplitude of the period itself and its four closest neighbors, divided by the average amplitude.

apq11Shimmer - Amplitude Perturbation Quotient for eleven points. It computes the average difference between the amplitude of a period and the average amplitude of the period itself and its ten closest neighbors, divided by the average amplitude.

localJitter - The average difference between consecutive periods, divided by the average period.

localabsJitter - The average absolute difference between consecutive periods in seconds.

rapJitter - Relative Average Perturbation, which is the average absolute difference between a period and the average of the period itself and its two neighboring periods, divided by the average period.

ppq5Jitter - Five-point Period Perturbation Quotient, which represents the average absolute difference between a period and the average of the period itself and its four closest neighbors, divided by the average period.
