# Supplementary material for: Impact of Audio Data Compression on Feature Extraction for Vocal Biomarker Detection: Validation Study
Source: JMIR Biomed Eng. 2024 Apr 15;9:e56246. doi: 10.2196/56246 (PMC11058552; doi:10.2196/56246)
Supplement: Multimedia Appendix 2 [file biomedeng_v9i1e56246_app2.docx]

**Table S1.** Wilcoxon sign rank test results (values) when comparing feature results for uncompressed and compressed audio formats. Formats were compressed with bitrates of 128kbps and 320kbps by MediaHuman (MH) encoders.

| Tool: MH | MP3 | | M4A | | WMA | |
| --- | --- | --- | --- | --- | --- | --- |
| Bitrate (kbps): | 128 | 320 | 128 | 320 | 128 | 320 |
| meanF0 | 72896531 (P=.003) | 73846763 (P=.13) | 74240183 (P=.36) | 74359827 (P=.46) | 74197798 (P=.34) | 74024988 (P=.22) |
| stdevF0 | 72714415 (P=.001) | 74023073 (P=.21) | 71448705 (P<.001) | 71620826 (P<.001) | 74800725 (P=.97) | 74643110 (P=.78) |
| maxF0 | 73460793 (P=.04) | 74802503 (P=.95) | 74379379 (P=.48) | 74457654 (P=.56) | 73296030 (P=.02) | 73299491 (P=.02) |
| minF0 | 74275241 (P=.40) | 73510920 (P=.04) | 73520707 (P=.044) | 73981377 (P=.19) | 72863368 (P=.003) | 73062245 (P=.007) |
| meanI | 0 (P<.001) | 0 (P<.001) | 4 (P<.001) | 3 (P<.001) | 19546072 (P<.001) | 15929054 (P<.001) |
| stdevI | 45823420 (P<.001) | 59067938 (P<.001) | 54987930 (P<.001) | 68237173 (P<.001) | 7943146 (P<.001) | 9216741 (P<.001) |
| hnr | 7961142 (P<.001) | 62490501 (P<.001) | 56393682 (P<.001) | 644709829 (P<.001) | 59273514 (P<.001) | 62635653 (P<.001) |
| localJitter | 67965695 (P<.001) | 74504067 (P=.61) | 74331237 (P=.44) | 73419211 (P=.03) | 73911245 (P=.16) | 73650776 (P=.07) |
| localabsJitter | 68060915 (P<.001) | 74630863 (P=.75) | 74214611 (P=.34) | 73425485 (P=.03) | 73792849 (P=.12) | 73530712 (P=.05) |
| rapJitter | 64592662 (P<.001) | 74742120 (P=.88) | 73354923 (P=.02) | 72535132 (P=.0004) | 73786445 (P=.11) | 73617815 (P=.07) |
| ppq5Jitter | 64796067 (P<.001) | 74293810 (P=.40) | 74269355 (P=.38) | 73340153 (P=.02) | 74094124 (P=.27) | 73979540 (P=.20) |
| localShimmer | 33648703 (P<.001) | 70977874 (P<.001) | 72519071 (P=.0004) | 74652668 (P=.77) | 72367192 (P=.0002) | 71927847 (P<.001) |
| localdbShimmer | 47596413 (P<.001) | 72714823 (P=.001) | 72254217(P=.00008) | 73630778 (P=.07) | 72324219 (P=.0001) | 72195329 (P<.001) |
| apq3Shimmer | 27606326 (P<.001) | 70109357 (P<.001) | 74529770 (P=0.63) | 72691873 (P=.001) | 71608695 (P<.001) | 71738653 (P<.001) |
| apq5Shimmer | 32795411 (P<.001) | 71339946 (P<.001) | 72114081 (P<.001) | 74383918 (P=.48) | 72451344 (P=.0003) | 71883982 (P<.001) |
| apq11Shimmer | 55163717 (P<.001) | 73030347 (P=.006) | 71033587 (P<.001) | 72411665 (P=.0002) | 73758060 (P=.10) | 73136236 (P=.01) |
| meanC0 | 0 (P<.001) | 3 (P<.001) | 7 (P<.001) | 57 (P<.001) | 10959845 (P<.001) | 8718004 (P<.001) |
| mean_mfcc1 | 500432 (P<.001) | 29145935 (P<.001) | 1134277 (P<.001) | 24460299 (P<.001) | 4397109 (P<.001) | 3266093 (P<.001) |
| mean_mfcc2 | 105894 (P<.001) | 13810950 (P<.001) | 304478 (P<.001) | 20396740 (P<.001) | 3826310 (P<.001) | 4098935 (P<.001) |
| mean_mfcc3 | 64792 (P<.001) | 6613090 (P<.001) | 61891 (P<.001) | 14924738 (P<.001) | 15353142 (P<.001) | 17791798 (P<.001) |
| mean_mfcc4 | 2225 (P<.001) | 495165 (P<.001) | 15 (P<.001) | 5788916 (P<.001) | 35860400 (P<.001) | 39277839 (P<.001) |
| mean_mfcc5 | 555 (P<.001) | 228000 (P<.001) | 8 (P<.001) | 2908662 (P<.001) | 44405793 (P<.001) | 47385726 (P<.001) |
| mean_mfcc6 | 2853 (P<.001) | 293479 (P<.001) | 97 (P<.001) | 2648191 (P<.001) | 45945199 (P<.001) | 48570078 (P<.001) |
| mean_mfcc7 | 4374 (P<.001) | 259095 (P<.001) | 0 (P<.001) | 2165525 (P<.001) | 52072396 (P<.001) | 56046839 (P<.001) |
| mean_mfcc8 | 67828 (P<.001) | 1897129 (P<.001) | 7415 (P<.001) | 5557699 (P<.001) | 56915385 (P<.001) | 61517799 (P<.001) |
| mean_mfcc9 | 421238 (P<.001) | 5822356 (P<.001) | 78292 (P<.001) | 8590959 (P<.001) | 54181774 (P<.001) | 57810850 (P<.001) |
| mean_mfcc10 | 935475 (P<.001) | 5538587 (P<.001) | 136425 (P<.001) | 8384500 (P<.001) | 43907142 (P<.001) | 45070583 (P<.001) |
| mean_mfcc11 | 1532113 (P<.001) | 1978334 (P<.001) | 191154 (P<.001) | 5378402 (P<.001) | 44242794 (P<.001) | 44073325 (P<.001) |
| mean_mfcc12 | 2381259 (P<.001) | 86156 (P<.001) | 421506 (P<.001) | 1477088 (P<.001) | 56720731 (P<.001) | 56454398 (P<.001) |
| sdC0 | 2839389 (P<.001) | 5605923 (P<.001) | 28201969 (P<.001) | 34049569 (P<.001) | 2878173 (P<.001) | 6040165 (P<.001) |
| sd_mfcc1 | 13833645 (P<.001) | 29849412 (P<.001) | 55398468 (P<.001) | 45646206 (P<.001) | 1037502 (P<.001) | 1569838 (P<.001) |
| sd_mfcc2 | 39567333 (P<.001) | 36305090 (P<.001) | 73224247 (P=.01) | 62307550 (P<.001) | 19699982 (P<.001) | 28525311 (P<.001) |
| sd_mfcc3 | 16237159 (P<.001) | 40216825 (P<.001) | 45658316 (P<.001) | 71285811 (P<.001) | 3082280 (P<.001) | 3176311 (P<.001) |
| sd_mfcc4 | 9834244 (P<.001) | 30316760 (P<.001) | 16136916 (P<.001) | 66134860 (P<.001) | 3362644 (P<.001) | 4155187 (P<.001) |
| sd_mfcc5 | 26007525 (P<.001) | 42183211(P<.001) | 50229554 (P<.001) | 73573049 (P=.05) | 3988591 (P<.001) | 5905696 (P<.001) |
| sd_mfcc6 | 3945249 (P<.001) | 22654871 (P<.001) | 9681668 (P<.001) | 60069995 (P<.001) | 4505268 (P<.001) | 5736397 (P<.001) |
| sd_mfcc7 | 18914032 (P<.001) | 58211484 (P<.001) | 52452256 (P<.001) | 66796439 (P<.001) | 3497723 (P<.001) | 5246994 (P<.001) |
| sd_mfcc8 | 1601897 (P<.001) | 28447629 (P<.001) | 12160357 (P<.001) | 60318783 (P<.001) | 944369 (P<.001) | 1514531 (P<.001) |
| sd_mfcc9 | 4151138 (P<.001) | 70227375 (P<.001) | 51662158 (P<.001) | 64751302 (P<.001) | 2259373 (P<.001) | 2802693 (P<.001) |
| sd_mfcc10 | 1133437 (P<.001) | 29289236 (P<.001) | 18701079 (P<.001) | 53114056 (P<.001) | 1303356 (P<.001) | 1679879 (P<.001) |
| sd_mfcc11 | 572377 (P<.001) | 39367473 (P<.001) | 22930716 (P<.001) | 64615841 (P<.001) | 1236436 (P<.001) | 2040728 (P<.001) |
| sd_mfcc12 | 322741 (P<.001) | 46792784 (P<.001) | 30419321 (P<.001) | 62883559 (P<.001) | 763925 (P<.001) | 1085478 (P<.001) |

Ppq, percent perturbation quotient; aapq, amplitude perturbation quotient; F0, fundamental frequency; HNR, harmonic to noise ratio; rap, relative average perturbation.

**Table S2.** Wilcoxon sign-rank test results (values) when comparing feature results for uncompressed and compressed audio formats. Formats were compressed with bitrates of 128kbps and 320kbps by WonderShare (WS) encoders.

| Tool: WS | MP3 | | M4A | | WMA | |
| --- | --- | --- | --- | --- | --- | --- |
| Bitrate (kbps): | 128 | 320 | 128 | 320 | 128 | 320 |
| meanF0 | 21719056 (P<.001) | 21669131 (P<.001) | 21536657 (P<.001) | 21487474 (P<.001) | 21139917 (P<.001) | 21138215 (P<.001) |
| stdevF0 | 56298039 (P<.001) | 56396736 (P<.001) | 57453888 (P<.001) | 57294762 (P<.001) | 56917144 (P<.001) | 56740123 (P<.001) |
| maxF0 | 19946511 (P<.001) | 19675794 (P<.001) | 21311111 (P<.001) | 21136399 (P<.001) | 21963492 (P<.001) | 21303157 (P<.001) |
| minF0 | 5331223 (P<.001) | 5332014 (P<.001) | 5726232 (P<.001) | 5643282 (P<.001) | 5736786 (P<.001) | 5547173 (P<.001) |
| meanI | 15460806 (P<.001) | 19461324 (P<.001) | 22219441 (P<.001) | 22325255 (P<.001) | 23369520 (P<.001) | 23532146 (P<.001) |
| stdevI | 21989838 (P<.001) | 21553093(P<.001) | 22315401 (P<.001) | 22296773 (P<.001) | 21562799 (P<.001) | 21620190 (P<.001) |
| hnr | 59976585 (P<.001) | 58036343 (P<.001) | 55002501 (P<.001) | 55090149 (P<.001) | 60537587 (P<.001) | 60526523 (P<.001) |
| localJitter | 52801871 (P<.001) | 52790332 (P<.001) | 49175250 (P<.001) | 49202443 (P<.001) | 50466917 (P<.001) | 50119722 (P<.001) |
| localabsJitter | 41340147 (P<.001) | 41258416 (P<.001) | 38176829 (P<.001) | 38222397 (P<.001) | 39234102 (P<.001) | 38903711 (P<.001) |
| rapJitter | 49579195 (P<.001) | 49497925 (P<.001) | 45975825 (P<.001) | 46085766 (P<.001) | 47099828 (P<.001) | 46844527 (P<.001) |
| ppq5Jitter | 47184837 (P<.001) | 47297399 (P<.001) | 43413718 (P<.001) | 43300806 (P<.001) | 43782957 (P<.001) | 43503869 (P<.001) |
| localShimmer | 38124621 (P<.001) | 36982130 (P<.001) | 34975540 (P<.001) | 34644919 (P<.001) | 35661404 (P<.001) | 35611610 (P<.001) |
| localdbShimmer | 33844265 (P<.001) | 33179980 (P<.001) | 31589050 (P<.001) | 31157273 (P<.001) | 32092151 (P<.001) | 32096698 (P<.001) |
| apq3Shimmer | 51455515 (P<.001) | 49259441 (P<.001) | 47252357 (P<.001) | 46831144 (P<.001) | 47621594 (P<.001) | 47505299 (P<.001) |
| apq5Shimmer | 48335645 (P<.001) | 47085583 (P<.001) | 45228888 (P<.001) | 44757318 (P<.001) | 45116346 (P<.001) | 44972134 (P<.001) |
| apq11Shimmer | 41015964 (P<.001) | 40773798 (P<.001) | 39846381 (P<.001) | 39499883 (P<.001) | 39604637 (P<.001) | 39458107 (P<.001) |
| meanC0 | 7239921 (P<.001) | 16636011 (P<.001) | 18808003 (P<.001) | 18789926 (P<.001) | 5432654 (P<.001) | 18761975 (P<.001) |
| mean_mfcc1 | 62459283 (P<.001) | 22849276 (P<.001) | 26789338 (P<.001) | 24872744 (P<.001) | 16165742 (P<.001) | 21972607 (P<.001) |
| mean_mfcc2 | 1134415 (P<.001) | 24751735 (P<.001) | 30128855 (P<.001) | 29918937 (P<.001) | 88457 (P<.001) | 31164584 (P<.001) |
| mean_mfcc3 | 6452209 (P<.001) | 44877069 (P<.001) | 44216495 (P<.001) | 41089395 (P<.001) | 206698 (P<.001) | 39718231 (P<.001) |
| mean_mfcc4 | 1023679 (P<.001) | 26139193 (P<.001) | 27123696 (P<.001) | 28673164 (P<.001) | 10684 (P<.001) | 29782955 (P<.001) |
| mean_mfcc5 | 7107425 (P<.001) | 29592964 (P<.001) | 26619896 (P<.001) | 23164723 (P<.001) | 342460 (P<.001) | 22534416 (P<.001) |
| mean_mfcc6 | 5537561 (P<.001) | 58532621 (P<.001) | 56147150 (P<.001) | 60133589 (P<.001) | 171454 (P<.001) | 61093010 (P<.001) |
| mean_mfcc7 | 244983 (P<.001) | 13925331 (P<.001) | 17881452 (P<.001) | 20370752 (P<.001) | 10921 (P<.001) | 21549202 (P<.001) |
| mean_mfcc8 | 6328162 (P<.001) | 37370185 (P<.001) | 36386691 (P<.001) | 30079192 (P<.001) | 265056 (P<.001) | 29277735 (P<.001) |
| mean_mfcc9 | 337376 (P<.001) | 10025085 (P<.001) | 12610523 (P<.001) | 14639100 (P<.001) | 39369 (P<.001) | 15210522 (P<.001) |
| mean_mfcc10 | 5035394 (P<.001) | 66431892 (P=0.004) | 68286401 (P=.12) | 61747282 (P<.001) | 429485 (P<.001) | 59567423 (P<.001) |
| mean_mfcc11 | 1353170 (P<.001) | 29593121(P<.001) | 36849246 (P<.001) | 42310430 (P<.001) | 168665 (P<.001) | 43658689 (P<.001) |
| mean_mfcc12 | 4832607 (P<.001) | 52292898 (P<.001) | 57190472 (P<.001) | 63253675 (P<.001) | 599242 (P<.001) | 64554535 (P<.001) |
| sdC0 | 20254533 (P<.001) | 13675352(P<.001) | 20088116 (P<.001) | 16833726 (P<.001) | 22146700 (P<.001) | 16776490 (P<.001) |
| sd_mfcc1 | 15307526(P<.001) | 35795029 (P<.001) | 36770994 (P<.001) | 36290931 (P<.001) | 29910092 (P<.001) | 35050489 (P<.001) |
| sd_mfcc2 | 52819493(P<.001) | 63040764 (P<.001) | 66586621 (P<.001) | 68991746 (P=.76) | 66307118 (P<.001) | 68613885 (P=.15) |
| sd_mfcc3 | 65703880 (P=.00008) | 17409258 (P<.001) | 18540176 (P<.001) | 16339859 (P<.001) | 36476259 (P<.001) | 16843891 (P<.001) |
| sd_mfcc4 | 30897208 (P<.001) | 51941281 (P<.001) | 59205089 (P<.001) | 54507712 (P<.001) | 50415968 (P<.001) | 56810005 (P<.001) |
| sd_mfcc5 | 62235076 (P<.001) | 61143702 (P<.001) | 63551389 (P<.001) | 64904097 (P<.001) | 58346974 (P<.001) | 64060433 (P<.001) |
| sd_mfcc6 | 9787614 (P<.001) | 54082133 (P<.001) | 55654894 (P<.001) | 61132896 (P<.001) | 25532315 (P<.001) | 61786439 (P<.001) |
| sd_mfcc7 | 61748315 (P<.001) | 38410910 (P<.001) | 48775630 (P<.001) | 44928575 (P<.001) | 68914276 (P=.37) | 45244395 (P<.001) |
| sd_mfcc8 | 46855350 (P<.001) | 26345983 (P<.001) | 34008568 (P<.001) | 27758058 (P<.001) | 69392175 (P=.90) | 27640050 (P<.001) |
| sd_mfcc9 | 56351524 (P<.001) | 39276816 (P<.001) | 47297637 (P<.001) | 42915366 (P<.001) | 68048731 (P=.02) | 42341785 (P<.001) |
| sd_mfcc10 | 18450087 (P<.001) | 52664060 (P<.001) | 54284071 (P<.001) | 45760550 (P<.001) | 45067075 (P<.001) | 44438518 (P<.001) |
| sd_mfcc11 | 60218764 (P<.001) | 27307844 (P<.001) | 36693786 (P<.001) | 30919043 (P<.001) | 61791640 (P<.001) | 29769629 (P<.001) |
| sd_mfcc12 | 35590391 (P<.001) | 39410281 (P<.001) | 45278759 (P<.001) | 38172199 (P<.001) | 64615050 (P<.001) | 37317343 (P<.001) |

Ppq, percent perturbation quotient; aapq, amplitude perturbation quotient; F0, fundamental frequency; HNR, harmonic to noise ratio; rap, relative average perturbation.

**Table S3.** Wilcoxon sign-rank test results (values) when comparing feature results for uncompressed and compressed audio formats. Formats were compressed with bitrates of 128kbps and 320kbps by FFmpeg encoder in python.

| Tool: FFmpeg | MP3 | | M4A | | WMA | |
| --- | --- | --- | --- | --- | --- | --- |
| Bitrate (kbps): | 128 | 320 | 128 | 320 | 128 | 320 |
| meanF0 | 74765466 (P=.89) | 74797684 (P=.93) | 74502901 (P=.61) | 74342334 (P=.45) | 74197798 (P=.34) | 74025114 (P=.22) |
| stdevF0 | 74007135 (P=.20) | 74642153 (P=.75) | 70609663 (P<.001) | 71820162 (P<.001) | 74800725 (P=.97) | 74643177 (P=.78) |
| maxF0 | 74789527 (P=.92) | 74809840 (P=.95) | 74547795 (P=.66) | 74562729 (P=.67) | 73296030 (P=.02) | 73299469 (P=.02) |
| minF0 | 73076009 (P=.007) | 74599834 (P=.70) | 73264516 (P=.02) | 74029527 (P=.22) | 72863368 (P=.003) | 73062184 (P=.007) |
| meanI | 1862 (P<.001) | 2262 (P<.001) | 0 (P<.001) | 0 (P<.001) | 19546072 (P<.001) | 15929015 (P<.001) |
| stdevI | 69288578 (P<.001) | 74320301 (P=.42) | 50858318 (P<.001) | 66548044 (P<.001) | 7943146 (P<.001) | 9216709 (P<.001) |
| hnr | 73465230 (P=.03) | 49284179 (P<.001) | 73157488 (P=.01) | 67706269 (P<.001) | 59273514 (P<.001) | 62636914 (P<.001) |
| localJitter | 73510116 (P=.04) | 73930980 (P=.16) | 73584244 (P=.06) | 73976299 (P=.19) | 73911245 (P=.16) | 73653913 (P=.07) |
| localabsJitter | 73650575 (P=.07) | 73847330 (P=.13) | 73905470 (P=.16) | 73916518 (P=.16) | 73792849 (P=.12) | 73534662 (P=.05) |
| rapJitter | 72935566 (P=.004) | 74482607 (P=.57) | 74159176 (P=.30) | 73554464 (P=.05) | 73786445 (P=.11) | 73618804 (P=.07) |
| ppq5Jitter | 73687799 (P=.08) | 74383225 (P=.48) | 73853347 (P=.13) | 73738420 (P=.09) | 74094124 (P=.26) | 73982984 (P=.20) |
| localShimmer | 68331873 (P<.001) | 73219994 (P=.01) | 61346674 (P<.001) | 70083082 (P<.001) | 72367192 (P=.0002) | 71928178 (P<.001) |
| localdbShimmer | 71779973 (P<.001) | 73670181 (P=.07) | 66293247 (P<.001) | 71854891 (P<.001) | 72324219 (P=.0001) | 72192637 (P<.001) |
| apq3Shimmer | 67769831 (P<.001) | 73889793 (P=.14) | 58512862 (P<.001) | 69540613 (P<.001) | 71608695 (P<.001) | 71743552 (P<.001) |
| apq5Shimmer | 68920169 (P<.001) | 74571122 (P=.67) | 59815767 (P<.001) | 69917073 (P<.001) | 72451344 (P=.0003) | 71884643 (P<.001) |
| apq11Shimmer | 71372237 (P<.001) | 74824821 (P=.97) | 67525393 (P<.001) | 72134227 (P<.001) | 73758060 (P=.10) | 73139473 (P=.01) |
| meanC0 | 46 (P<.001) | 37319 (P<.001) | 0 (P<.001) | 0 (P<.001) | 10959845 (P<.001) | 8717983 (P<.001) |
| mean_mfcc1 | 48 (P<.001) | 28546528 (P<.001) | 4341206 (P<.001) | 64399070 (P<.001) | 4397109 (P<.001) | 3266070 (P<.001) |
| mean_mfcc2 | 0 (P<.001) | 14869259 (P<.001) | 1300118 (P<.001) | 9943105 (P<.001) | 3826310 (P<.001) | 4098983 (P<.001) |
| mean_mfcc3 | 0 (P<.001) | 9484205 (P<.001) | 92077 (P<.001) | 42293342 (P<.001) | 15353142 (P<.001) | 17792235 (P<.001) |
| mean_mfcc4 | 30 (P<.001) | 1393190 (P<.001) | 301 (P<.001) | 64323021 (P<.001) | 35860400 (P<.001) | 39278129 (P<.001) |
| mean_mfcc5 | 960 (P<.001) | 945113 (P<.001) | 342 (P<.001) | 70814440 (P<.001) | 44405793 (P<.001) | 47386113 (P<.001) |
| mean_mfcc6 | 1132 (P<.001) | 3274273 (P<.001) | 1331 (P<.001) | 64826048 (P<.001) | 45945199 (P<.001) | 48570250 (P<.001) |
| mean_mfcc7 | 367 (P<.001) | 562564 (P<.001) | 88 (P<.001) | 70348326 (P<.001) | 52072396 (P<.001) | 56046293 (P<.001) |
| mean_mfcc8 | 0 (P<.001) | 2452137 (P<.001) | 48875 (P<.001) | 45403666 (P<.001) | 56915385 (P<.001) | 61517804 (P<.001) |
| mean_mfcc9 | 0 (P<.001) | 6464048 (P<.001) | 81690 (P<.001) | 58602429 (P<.001) | 54181774 (P<.001) | 57811037 (P<.001) |
| mean_mfcc10 | 0 (P<.001) | 5946797 (P<.001) | 79859 (P<.001) | 44038368 (P<.001) | 43907142 (P<.001) | 45070747 (P<.001) |
| mean_mfcc11 | 0 (P<.001) | 1884382 (P<.001) | 28296 (P<.001) | 53495083 (P<.001) | 44242794 (P<.001) | 44073657 (P<.001) |
| mean_mfcc12 | 7 (P<.001) | 1466221 (P<.001) | 69 (P<.001) | 60721586 (P<.001) | 56720731 (P<.001) | 56454397 (P<.001) |
| sdC0 | 34627917 (P<.001) | 3790500 (P<.001) | 14126038 (P<.001) | 27559013 (P<.001) | 2878173 (P<.001) | 6040093 (P<.001) |
| sd_mfcc1 | 20387947 (P<.001) | 41926274 (P<.001) | 73939384 (P=.17) | 66787200 (P<.001) | 1037502 (P<.001) | 1569905 (P<.001) |
| sd_mfcc2 | 4730572 (P<.001) | 62944610 (P<.001) | 71966358 (P<.001) | 74640494 (P=.76) | 19699982 (P<.001) | 28525137 (P<.001) |
| sd_mfcc3 | 53757361 (P<.001) | 74804766 (P=.94) | 67956480 (P<.001) | 72581436 (P=.0006) | 3082280 (P<.001) | 3176114 (P<.001) |
| sd_mfcc4 | 30431019 (P<.001) | 68249743 (P<.001) | 26469726 (P<.001) | 61895712 (P<.001) | 3362644 (P<.001) | 4155157 (P<.001) |
| sd_mfcc5 | 11535114 (P<.001) | 67636765 (P<.001) | 66395417 (P<.001) | 71194029 (P<.001) | 3988591 (P<.001) | 5905725 (P<.001) |
| sd_mfcc6 | 36344662 (P<.001) | 65743358 (P<.001) | 18901822 (P<.001) | 70270152 (P<.001) | 4505268 (P<.001) | 5736512 (P<.001) |
| sd_mfcc7 | 3372988 (P<.001) | 63945386 (P<.001) | 71875022 (P<.001) | 68437255 (P<.001) | 3497723 (P<.001) | 5247284 (P<.001) |
| sd_mfcc8 | 43474335 (P<.001) | 64606773 (P<.001) | 23997131 (P<.001) | 68092540 (P<.001) | 944369 (P<.001) | 1514042 (P<.001) |
| sd_mfcc9 | 7194406 (P<.001) | 58831395 (P<.001) | 72468718 (P=.0003) | 74500648 (P=.60) | 2259373 (P<.001) | 2802720 (P<.001) |
| sd_mfcc10 | 66822115 (P<.001) | 60520072 (P<.001) | 32536986 (P<.001) | 71215799 (P<.001) | 1303356 (P<.001) | 1679837 (P<.001) |
| sd_mfcc11 | 5326208 (P<.001) | 61558948 (P<.001) | 36581076 (P<.001) | 74802220 (P=.95) | 1236436 (P<.001) | 2040992 (P<.001) |
| sd_mfcc12 | 27092468 (P<.001) | 60688874 (P<.001) | 38651964 (P<.001) | 72439863 (P=.0003) | 763925 (P<.001) | 1085357 (P<.001) |

Ppq, percent perturbation quotient; aapq, amplitude perturbation quotient; F0, fundamental frequency; HNR, harmonic to noise ratio; rap, relative average perturbation.
